# Contribution of White matter Hyperintensities Lesion Burden to Cognitive Impairment in Older Adults with Epilepsy

**DOI:** 10.1002/alz.090744

**Published:** 2025-01-09

**Authors:** Anny Reyes, Divya Prabhakaran, Nikdokht Farid, Vineet Punia, Aaron F Struck, Irene Wang, Lisa Ferguson, Dace Almane, Jana Jones, Robyn M Busch, Bruce P Hermann, Carrie R McDonald

**Affiliations:** ^1^ University of California, San Diego, San Diego, CA USA; ^2^ Cleveland Clinic, Cleveland, OH USA; ^3^ University of Wisconsin School of Medicine and Public Health, Madison, WI USA; ^4^ Department of Neurology, University of Wisconsin‐Madison School of Medicine and Public Health, Madison, WI USA

## Abstract

**Background:**

Older adults with epilepsy represent the largest and fastest‐growing segment of individuals with epilepsy and harbor risk factors for pathological aging, including cerebrovascular risk factors (CVRFs) and Alzheimer’s disease (AD)‐related pathology. In fact, several community‐based studies have reported up to a 3‐fold increased risk for dementia including AD among individuals with epilepsy. Despite this, identification of risk factors for AD and related dementias (ADRD) remains largely unexplored in epilepsy, which has critical implications for patient care and dementia risk stratification. Here, we examine the relationship between white matter hyperintensities (WMH) as a marker for vascular brain injury, a common contributor to the ADRD spectrum (i.e., biomarker of non‐AD co‐pathology), and sociodemographic, clinical, and cognitive profiles in older adults with epilepsy.

**Method:**

Forty‐nine older adults with epilepsy without dementia (average age=66.6, average education=15.1, 52.8% female, 86.8% non‐Hispanic white) completed cognitive testing, a comprehensive lipid panel, and MRI that included 3D FLAIR. The 3D FLAIR was analyzed to quantify WMH lesion burden (LB) using the LesionQuant software. Stepwise regressions were used to examine the contribution of sociodemographic, clinical, and vascular risk factors to WMH‐LB. The association between cognitive scores and WMH‐LB was analyzed with Spearman rho.

**Result:**

Older age, male sex, non‐White race, hypertension, and diabetes were associated with increased periventricular (adjusted R2= .518, p=.016) and whole brain WMH‐LB (adjusted R2= .456, p=.025). Smoking was associated with increased juxtacortical WMH‐LB (β= .684, p=.025). Higher juxtacortical WMH‐LB was associated with poorer performance on measures of visual immediate (rho=‐.374, p=.010) and delayed memory (rho=‐.408, p=.004), delayed list recall (rho=‐.363, p=.011), processing speed (rho=‐.364, p=.010), confrontation naming (rho=‐.314, p=.010), and lower scores on the MoCA (rho=‐.350, p=.014) and more errors on the ADAS‐Cog (rho=.372, p=.008).

**Conclusion:**

In a group of older adults with epilepsy, greater WMH burden was associated with a worse cognitive profile and with the presence of CVRFs. The etiology of dementia in epilepsy remains understudied, and here, we provide evidence for WMH burden as a potential contributing factor to cognitive decline in this population. Older adults with epilepsy with evidence of vascular pathology would benefit from targeted interventions focused on the management of modifiable vascular risk factors.

